# Kidney Outcomes in Patients With Hereditary Transthyretin Amyloid Nephropathy Treated With Transthyretin Stabilizers And Gene-Silencer Therapies

**DOI:** 10.1016/j.ekir.2025.03.029

**Published:** 2025-03-24

**Authors:** Julien Dang, Justine Solignac, Sophie Ferlicot, Charlotte Mussini, Frank Bridoux, Antoine Huart, Marie Essig, Delphine Campigli, Mickaël Bobot, Laurent Daniel, Thibaud Damy, Violaine Planté-Bordeneuve, Hamza Sakhi, Céline Labeyrie, Cécile Cauquil, Andoni Echaniz-Laguna, Ilias Kounis, Anissa Moktefi, Perrine Devic, Sarah Mouawad, Albane Brodin-Sartorius, Renaud Snanoudj, Mohamad Zaidan, Noémie Jourde-Chiche, Vincent Audard

**Affiliations:** 1 Assistance Publique des Hôpitaux de Paris, Hôpital Ambroise Paré, Service de Néphrologie et Dialyse, Boulogne-Billancourt, France; 2 Université Versailles-Saint-Quentin, UFR Simone-Veil Santé, INSERM U1179, Montigny-le-Bretonneux, France; 3 AP-HP, Hôpital de Bicêtre, Service de Néphrologie et Transplantation, Le Kremlin-Bicêtre, France; 4 Aix-Marseille Université, C2VN, INSERM, INRAE, Marseille, France - AP-HM, Hôpital de la Conception, Service de Néphrologie et Transplantation rénale, Marseille, France; 5 AP-HP, Hôpital de Bicêtre, Service d’Anatomie Pathologique, Le Kremlin-Bicêtre, France; 6 Hôpital de Poitiers, Service de Néphrologie, Hémodialyse et Transplantation rénale, Poitiers, France; 7 Service de Néphrologie et Transplantation d’organes, Hôpital Rangueil, Toulouse, France; 8 Service d’Anatomie Pathologique, Hôpital de la Conception, Marseille, France; 9 Service de Cardiologie, AP-HP, Hôpitaux Universitaires Henri Mondor, Créteil, France; 10 Service de Neurologie, AP-HP, Hôpitaux Universitaires Henri Mondor, Créteil, France; 11 Service de Néphrologie et Transplantation, AP-HP, Hôpitaux Universitaires Henri Mondor, Créteil, France; 12 Université Paris Est-Créteil, INSERM U955, Institut Mondor de recherche Biomédicale, Créteil, France; 13 Service de Neurologie, AP-HP, Hôpital de Bicêtre, Le Kremlin-Bicêtre, France; 14 Centre Hépato-Biliaire, AP-HP, Hôpital Paul-Brousse, Villejuif, France; 15 Service d’Anatomie Pathologique, AP-HP, Hôpitaux Universitaires Henri Mondor, Créteil, France; 16 Service de Neurologie, Hospices Civils de Lyon, Hôpital Lyon Sud, Lyon, France

**Keywords:** gene silencing, genetic kidney disease, glomerular disease, hereditary transthyretin amyloidosis, nephrotic syndrome, systemic amyloidosis

## Abstract

**Introduction:**

Kidney involvement is underestimated in patients with hereditary transthyretin amyloidosis (ATTRv), and few data are available about the renal outcomes of patients treated with targeted therapies.

**Methods:**

Patients with ATTRv nephropathy (ATTRv-N) from 6 French referral centers were retrospectively included. The evolution of estimated glomerular filtration rate (eGFR) and proteinuria, and the specific treatments of ATTRv were collected. Renal survival was assessed by using a renal composite end point, including an eGFR decline > 50% from baseline and/or dialysis requirement.

**Results:**

Twenty-three patients (70% female) with a median age at ATTRv-N diagnosis of 50 (interquartile range [IQR]: 37–63) years were included. Baseline eGFR was 60 (39–83) ml/min per 1.73 m^2^. Median urine protein-to-creatinine ratio (UPCR) was 100 (IQR: 20–240) mg/mmol. ATTRv-N was documented by kidney biopsy in 20 of 23 patients (87%). Eleven patients were treated with the transthyretin (TTR) stabilizer, tafamidis; 6 patients with a small interfering RNA (siRNA); 4 with exclusive orthotopic liver transplantation (OLT); whereas 2 received no specific treatment. After a median follow-up of 5.8 (IQR: 3.3–18.6) years, all patients with OLT or no treatment had a progressive eGFR decline, requiring dialysis in 3 of 6 patients. Among patients treated with tafamidis, 8 of 11 (73%) had a progressive eGFR decline, requiring dialysis in 1 patient; and proteinuria was either stable or increasing over time in all patients. All patients who received siRNA therapy had stable or improving eGFR. Renal survival was 44%, 44%, and 100% at 60 months for the patients with exclusive OLT or no treatment, TTR stabilizers, and siRNA, respectively (*P* = 0.12). Four patients with baseline nephrotic syndrome or high-grade proteinuria, including 3 patients resistant to tafamidis, responded dramatically to siRNA therapy, with a fast, complete, and sustained remission of proteinuria within 1 year.

**Conclusion:**

Our study highlights the underrecognized risk of chronic kidney disease (CKD) and end-stage kidney disease in ATTRv and suggests that siRNA could be a promising therapeutic option for the stabilization of kidney function.

## Graphical abstract

**Figure:**
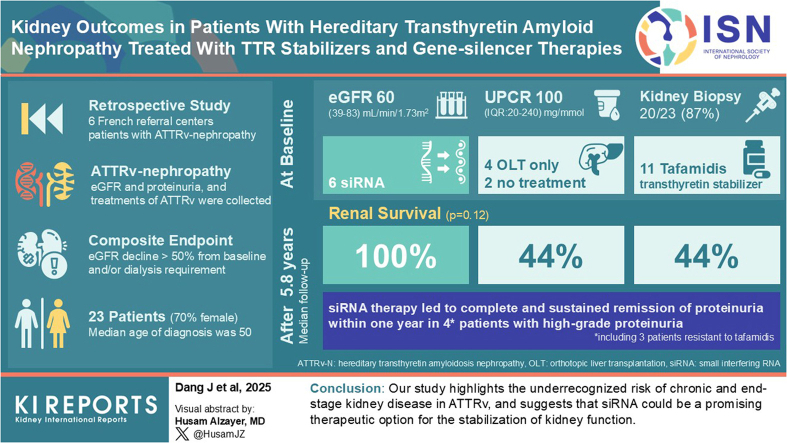

See Commentary on Page 1622

ATTR is a progressive, systemic, and ultimately fatal disease encompassing hereditary ATTR (ATTRv, v for variant) and wild-type (or senile) ATTR.^1^^,^^2^ ATTRv results from genetic variants in the *TTR* gene, whereas wild-type ATTR is associated with aging mechanisms. Both conditions are caused by accumulation of misfolded TTR protein into toxic amyloid fibrils that deposit in multiple organs and tissues, including the heart, peripheral nerves, and the gastrointestinal tract. Kidney involvement has been largely overlooked in clinical practice; however, we and others, have recently pointed out the underestimated prevalence of CKD among patients with ATTRv, either caused by direct amyloid deposition or indirect mechanisms.^3^, ^4^, ^5^, ^6^

Improvements in care over the past 10 years because of new effective and specific treatments, such as TTR stabilizers and gene silencers (siRNA, or antisense oligonucleotides) have resulted in a longer lifespan for patients with ATTRv, but may be associated with a higher cumulative risk of renal involvement. To date, potential benefits of targeted therapies of ATTRv on renal parameters remain to be determined.

In the present study, we sought to investigate the evolution of renal parameters in patients diagnosed with ATTRv-N, defined as biopsy-proven or very likely direct kidney involvement by TTR amyloid deposits, recruited in 6 French referral centers for amyloidosis.

## Methods

### Study Population and Data Collection

All adult patients with ATTRv-N diagnosed at the rare disease referral centers for amyloidosis (Bicêtre, Henri Mondor, Marseille, Poitiers, Toulouse, and Lyon University Hospitals), from December 1995 to June 2024, were considered for inclusion in this retrospective observational study. Patients were identified through the electronic database from the pathology departments. We defined ATTRv-N as presence of TTR amyloid deposits on kidney biopsy, or presence of persisting UPCR ≥ 100 mg/mmol without diabetes mellitus or other cause of proteinuria-associated nephropathy. Demographic, clinical, and biological findings were collected from medical charts. Patients with missing data regarding renal parameters (eGFR and/or UPCR) and/or with a follow-up < 6 months were excluded from the study. The study was performed in accordance with the Declaration of Helsinki and was approved by our local ethics committee (Créteil) and by the French Comité National de l’Informatique et des Libertés (CNIL number 2234409v0).

### Positive Diagnosis Criteria for ATTRv

Tissues with Congo red positive deposits were systematically stained with antibodies against TTR and serum amyloid A protein by immunohistochemistry and immunoglobulin light chains by immunofluorescence. The diagnosis of ATTRv included the presence of TTR-labeled amyloid deposits, together with the identification of a disease-causing *TTR* variant by gene sequencing. Alternatively, ATTRv could be diagnosed on the basis of the identification of a *TTR* variant in cases with established nonbiopsy diagnostic criteria (strong cardiac fixation on ^99m^technetium-bisphosphonate bone scintigraphy in patients with no evidence of monoclonal gammopathy).^7^ All patients included in this study had a genetic diagnosis of ATTRv, through direct sequencing of the *TTR* gene full coding region, or of a specific exon when a mutation had been identified in the family, as previously described.^8^

### Pathological Examination

In patients who underwent kidney biopsy, the abundance of glomerular, arterial, arteriolar, and interstitial amyloid deposition was graded semiquantitatively as follows: 0, absent; 1, mild; 2, moderate; and 3, abundant. Interstitial fibrosis of the cortical surface was scored according to the Banff 2019 working classification,^9^ that is, grade 0 (< 5%), grade 1 (6%–25%), grade 2 (26%–50%), and grade 3 (> 50%).

### Kidney Function Assessment

eGFR was calculated from the serum creatinine using the CKD—Epidemiology Collaboration formula.^10^ Significant proteinuria was defined by a UPCR ≥ 50 mg/mmol on a spot urine sample. eGFR and UPCR were collected at the time of ATTRv-N diagnosis and during follow-up. Renal survival was assessed by a composite renal end point (eGFR decline > 50% from baseline and/or dialysis requirement) and censored at death, loss to follow-up, or OLT for patients who received medical targeted therapies.

### Statistical Analysis

Continuous variables were described using median values (IQR 25-75), whereas categorical variables were given as counts and percentages. Frequency differences for qualitative variables were compared in χ2 tests. Nonparametric tests were used for quantitative variables. All tests were 2-tailed and *P* value < 0.05 was considered as statistically significant. Kaplan-Meier curves were used to compare renal survival between the 3 groups. Statistical analyses were performed using GraphPad Prism 8.0 (La Jolia, CA).

## Results

### Clinical Characteristics at the Time of ATTRv-N Diagnosis

From December 1995 to June 2024, 36 patients were diagnosed with ATTRv-N in 6 French referral centers for amyloidosis. Renal follow-up data was missing for 13 patients, who were excluded. Finally, 23 patients (16 women [70%] and 7 men [30%]) were included, with a median age of 50 (IQR: 37–63) years. Baseline characteristics are summarized in Table 1. The underlying *TTR* variant was V30M in 19 of 23 patients (83%) (including 1 patient with no congenital *TTR* mutation but who developed ATTRv symptoms 10 years after receiving a domino liver transplant from a V30M donor), V122I in 3 patients (13%), and S50R in 1 patient (4%). Eleven patients were born in Portugal, 8 in metropolitan France, 1 in the French West Indies (Guadeloupe), and 3 in Africa (Congo, Cameroon, and Ghana). Renal symptoms had led to the diagnosis of ATTRv in 7 of 23 patients (30%). Age at first symptoms of ATTRv was 43 (IQR: 31–54) years, and duration of these symptoms (peripheral neuropathy in 65% of cases) at the time of ATTRv-N diagnosis was 53 (IQR: 14–84) months. Eight patients were diagnosed with ATTRv-N while receiving a specific treatment for ATTRv; besides the domino liver recipient, 4 other patients had received an OLT from a cadaveric donor for the treatment of ATTRv, and 4 additional patients were treated with a TTR stabilizer (tafamidis).

**Table 1:** Clinical characteristics of patients at the time of diagnosis of ATTRv-N

Variables	All patients (*N* = 22)
Demographic characteristics	Median (IQR) or *n* (%)
Age (yrs)	50 (37–63)
Female	16/23 (70)
BMI (kg/m^2^)	21.1 (17.7–23.1)
Medical history	
Hypertension	9/23 (39)
Diabetes mellitus	0/23 (0)
Dyslipidemia	3/23 (13)
Smoking	2/23 (9)
Treatment at baseline	
ACE inhibitor/ARB	9/23 (39)
ATTRv characteristics	
Mutations	
V30M	18/23 (78)
V122I	3/23 (13)
S50R	1/23 (4)
Domino liver transplantation	1/23 (4)
Familial history of ATTR	17/23 (74)
Age at first symptoms	43 (31-54)
Duration of symptoms before ATTRv-N diagnosis (months)	53 (14-84)
Associated symptoms	
Peripheral neuropathy	22/23 (96)
Cardiac involvement	16/23 (70)
Hypertrophic cardiomyopathy	10/23 (44)
LVEF < 45%	2/23 (9)
Atrial fibrillation	5/23 (22)
PM or ICD	13/23 (57)
Orthostatic hypotension	11/23 (48)
Gastrointestinal disorder	11/23 (48)
Bladder dysfunction	4/23 (17)
Symptoms revealing ATTRv	
Neurological	15/23 (65)
Cardiac	1/23 (4)
Renal	7/23 (30)
Renal features at baseline	
eGFR (ml/min per 1.73 m^2^)	60 (39-83)
eGFR < 60 ml/min per 1.73 m^2^	13/23 (57)
CKD stage 1	3/23 (13)
CKD stage 2	8/23 (35)
CKD stage 3	10/23 (44)
CKD stage 4	2/23 (9)
UPCR (mg/mmol)	100 (20-240)
Significant proteinuria^a^	16/23 (70)
eGFR < 60 ml/min per 1.73 m^2^ and/or significant proteinuria	19/23 (83)
Microscopic hematuria	2/23 (9)
Biopsy-proven ATTRv-N	20/23 (87)

ACE, angiotensin converting enzyme; ARB, angiotensin II receptor blocker; ATTRv, hereditary transthyretin amyloidosis; ATTRv-N, ATTRv nephropathy; BMI, body mass index; CKD, chronic kidney disease; CKD-EPI, Chronic Kidney Disease - Epidemiology collaboration; eGFR, estimated glomerular filtration rate by CKD-EPI; ICD, implantable cardioverter defibrillator; LVEF, left ventricular ejection fraction; PM, pacemaker; UPCR, urinary protein-to creatinine-ratio.

Data are presented as *n* (percentage) of patients or median (interquartile range).

a Urine protein-to-creatinine ratio > 50 mg/mmol.

### Kidney Function and Biopsy Findings at the Time of ATTRv-N Diagnosis

Renal parameters are detailed in Table 1, Table 2. Median baseline eGFR was 60 (39–83) ml/min per 1.73 m^2^ and was < 60 ml/min per 1.73 m^2^ in 13 of 23 patients (57%). Median UPCR was 100 (IQR: 20–240) mg/mmol and was > 50 mg/mmol in 16 of 23 patients (70%). Overall, 83% (19/23) of patients displayed either an eGFR < 60 ml/min per 1.73 m^2^ and/or significant proteinuria at the time of ATTRv-N diagnosis. Microscopic hematuria was documented in 2 patients only. The diagnosis of ATTRv-N was biopsy-proven in 20 of 23 patients (87%). Kidney biopsy was performed in 14 of 20 cases for significant proteinuria and/or eGFR < 60 ml/min per 1.73 m^2^, and otherwise for systemic evaluation before considering liver transplantation alone. All kidney biopsies showed significant TTR amyloid deposits by immunohistochemistry analyses. Pathological findings are summarized in Table 2. The three remaining patients with very likely ATTRv-N had a UPCR > 100 mg/mmol without any other obvious cause of proteinuria–associated kidney disease than ATTRv.

**Table 2:** Detailed clinical and pathological characteristics of patients with ATTRv-N at the time of diagnosis

Treatment group	Demographic characteristics				Past Medical History	ATTRv characteristics & kidney involvement											
	Patient	Gender^a^	Age	Place of birth	HBP	Mutation	Age at first symptoms	LVEF (%)	Glomeruli^c^	Arteries^c^	Arterioles^c^	Interstitium^c^	Sclerotic glomeruli	Interstitial fibrosis^d^^,^^e^	eGFR (mL/min per 1.73 m^2^)	UPCR (mg/mmol)	Treatment before ATTRv-N
1: OLT or no treatment	1	F	35	Portugal	−	V30M	34	65	3	3	3	2	5/18	1	83	50	-
	2	F	69	France	+	V30M	66	ND	NA	1	1	2	0/0	NA	76	350	-
	3	M	29	Portugal	−	V30M	25	70	1	0	1	0	0/10	0	112	10	-
	4	F	44	Portugal	−	V30M	42	56	NA	NA	0	2	0/0	NA	108	20	-
	5	F	50	Portugal	+	V30M	31	60	2	3	3	3	3/11	2	41	180	-
	6	M	41	Ghana	+	V122I	37	65	NB	NB	NB	NB	NB	NB	40	100	-
2: TTR stabilizer	7	F	67	France	−	Domino	65	60	1	NA	1	2	13/19	3	29	250	Tafamidis
	8	F	52	Portugal	+	V30M	49	55	3	3	3	3	10/24	1	28	100	-
	9	F	47	France	+	V30M	46	65	1	NA	0	3	1/9	0	75	100	OLT
	10	F	34	Portugal	−	V30M	27	43	1	1	1	3	1/5	0	107	60	OLT
	11	M	52	Portugal	+	V30M	32	67	1	NA	0	1	15/19	3	47	20	OLT
	12	F	55	France	−	V30M	50	65	1	0	2	2	2/5	1	50	50	Tafamidis
	13	M	42	Portugal	−	V30M	20	76	3	1	3	3	6/15	0	60	220	-
	14	M	37	Portugal	−	V30M	30	67	3	0	3	3	7/35	2	74	20	-
	15	M	60	France	+	V30M	54	65	NB	NB	NB	NB	NB	NB	36	240	-
	16	F	65	France^b^	−	V122I	50	45	0	0	0	1	2/17	1	24	10	-
	17	F	75	Cameroon	−	V122I	74	20	NB	NB	NB	NB	NB	NB	31	150	-
3: Gene silencer	18	F	37	Portugal	−	V30M	31	49	1	0	1	0	0/14	0	80	<10	OLT
	19	F	63	France	−	V30M	61	66	3	NA	2	3	1/10	1	57	800	-
	20	F	37	Portugal	−	V30M	37	65	2	0	1	0	1/8	0	100	290	-
	21	M	67	Congo	+	V30M	65	55	2	0	1	0	0/14	1	72	150	Tafamidis
	22	F	54	France	−	S50R	49	60	0	0	0	1	0/8	0	39	10	Tafamidis
	23	F	43	France	−	V30M	43	65	3	2	2	0	2/12	2	85	265	-

ATTRv, hereditary transthyretin amyloidosis; ATTRv-N, ATTRv nephropathy; DM, diabetes mellitus; eGFR, estimated glomerular filtration rate according to CKD-EPI (Chronic Kidney Disease Epidemiology collaboration); HBP, high blood pressure; NA, not applicable; NB, no kidney biopsy; ND, no data; OLT, orthotopic liver transplantation; UPCR, urinary protein-to-creatinine ratio.

a Female (F) or Male (M).

b Guadeloupe.

c Amyloid deposition score = 0, absent; 1, mild; 2, moderate; 3 severe.

d NA if no cortical fragment.

e 0 = <5%, 1 = 6%–25%, 2 = 26%–50%, 3 = >50% of the cortical surface.

### Renal Outcomes on Specific Therapies

**Figure 1:**
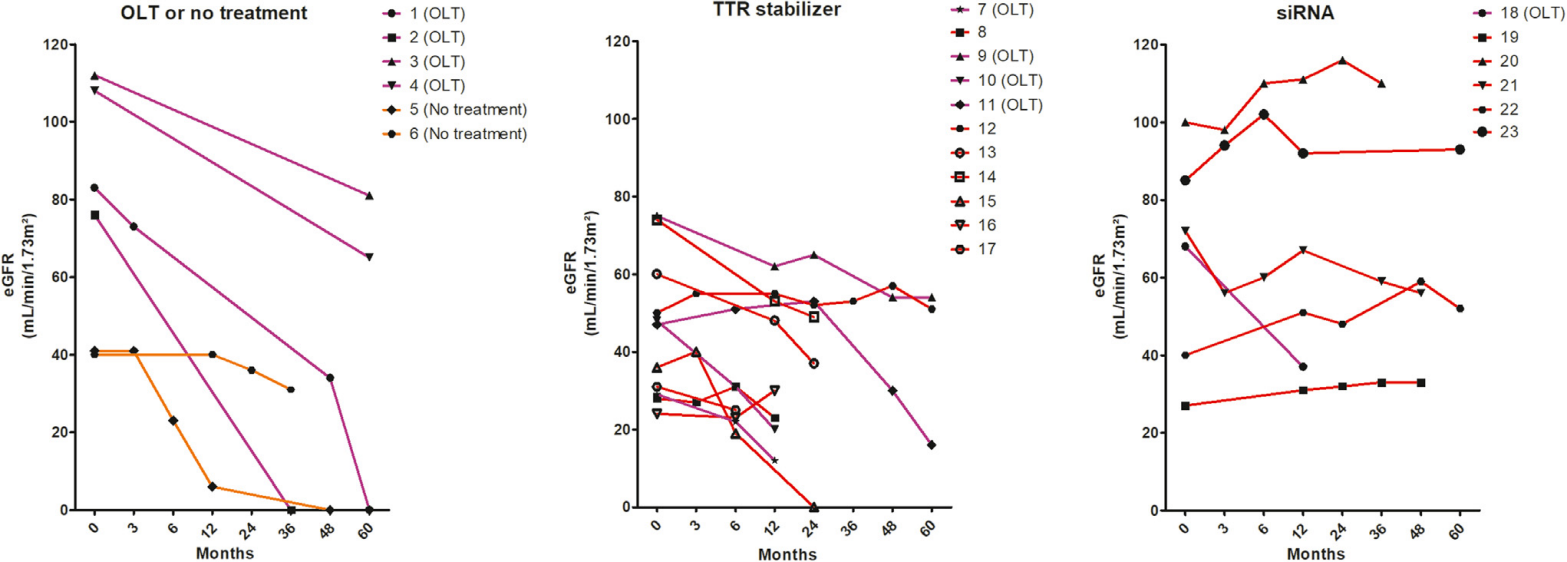
Evolution of eGFR in patients with ATTRv-N according to specific treatment. Evolution of estimated glomerular filtration rate (eGFR) (CKD-EPI) in patients with hereditary transthyretin amyloidosis (ATTRv) and related nephropathy (ATTRv-N) according to patients’ treatment: no treatment (orange lines) or orthotopic liver transplantation (OLT, purple lines) only (left panel), TTR stabilizer only (central panel, red lines), or siRNA only (right panel, red lines). Patients with liver transplantation before specific treatment initiation are highlighted (central and right panel, purple lines). ATTRv-N, ATTRv nephropathy; CKD-EPI, Chronic Kidney Disease - Epidemiology Collaboration formula; siRNA, small interfering RNA; TTR, transthyretin.

**Figure 2:**
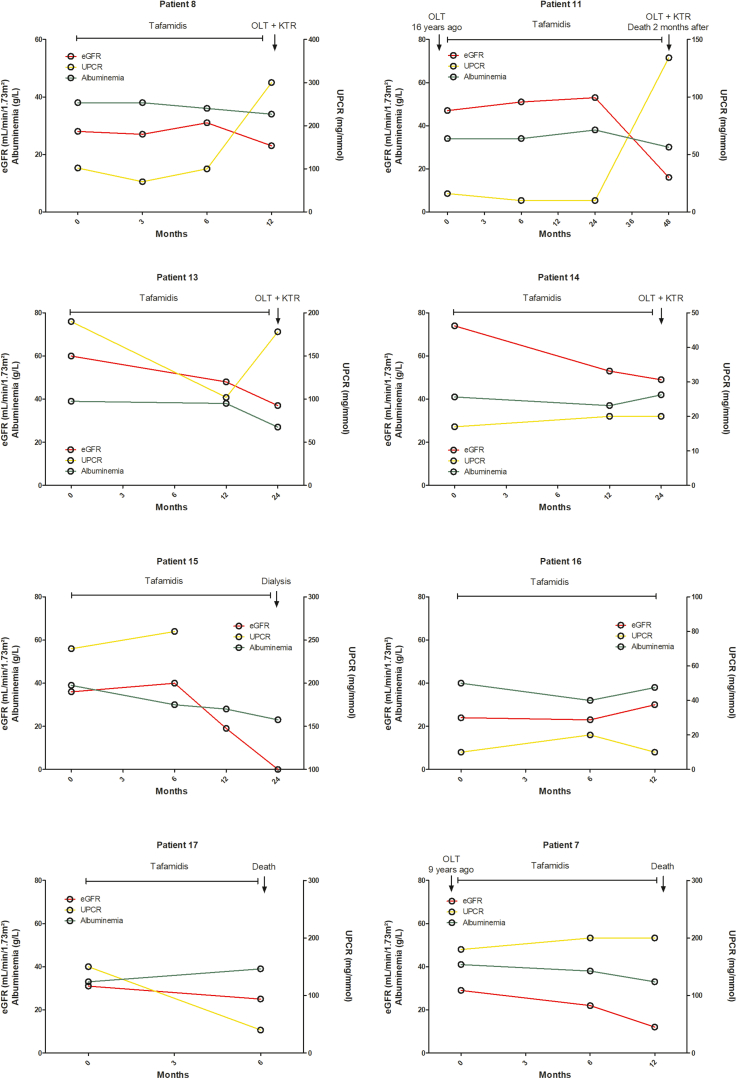
Evolution of kidney function with tafamidis in patients with ATTRv-N. Evolution of estimated glomerular filtration rate (eGFR) (CKD-EPI) (red lines), serum albumin levels (green lines) and urine protein-to-creatinine ratio (UPCR) (yellow lines) in patients with hereditary transthyretin amyloid nephropathy (ATTRv-N), after treatment with tafamidis. Data were censored at the time of orthotopic liver transplantation, dialysis, loss to follow-up or death. ATTRv-N, ATTRv nephropathy; CKD-EPI, Chronic Kidney Disease - Epidemiology Collaboration formula; siRNA, small interfering RNA.

**Figure 3:**
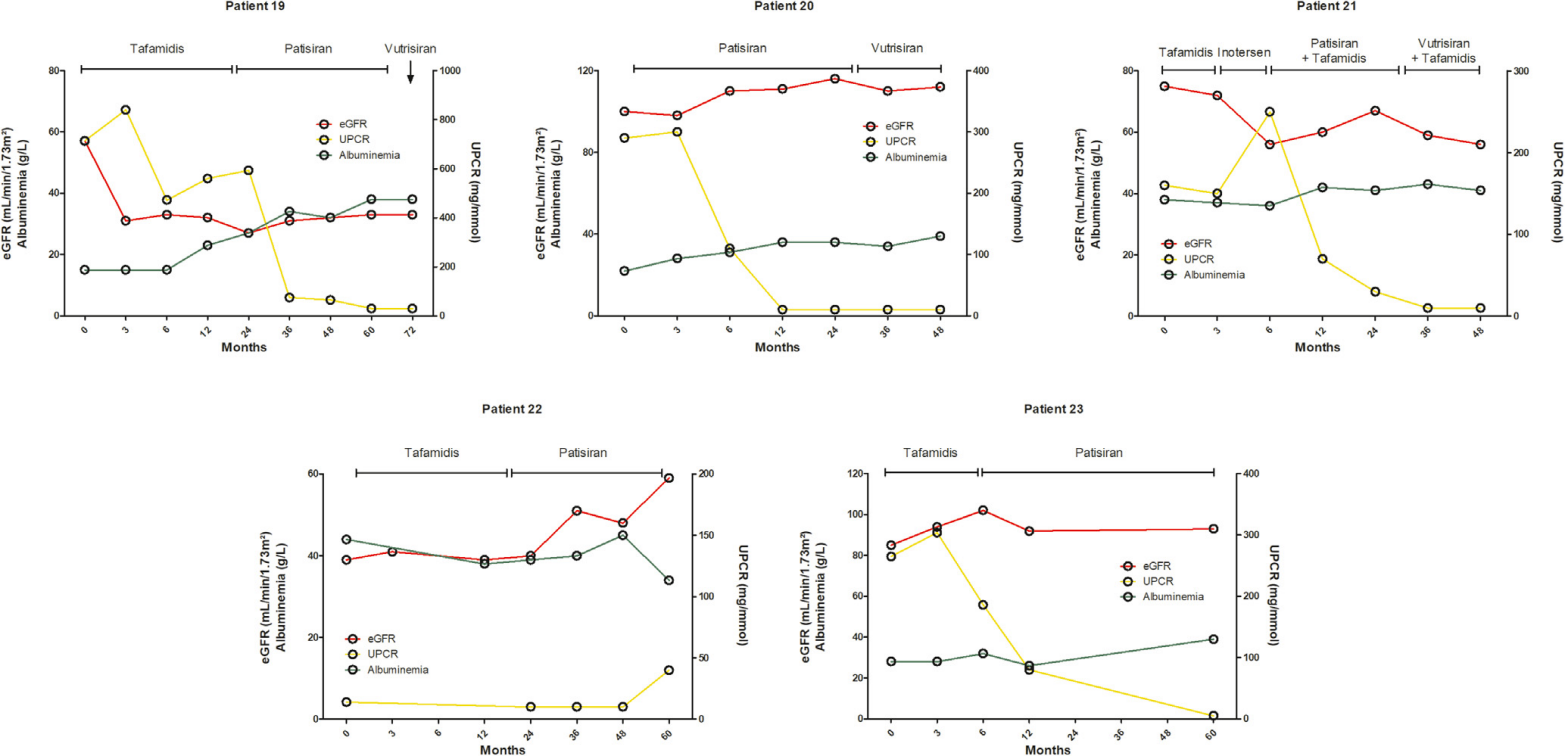
Evolution of kidney function under siRNA in patients with ATTRv-N. Evolution of estimated glomerular filtration rate (eGFR) (CKD-EPI) (red lines), serum albumin levels (green lines) and urine protein-to-creatinine ratio (UPCR) (yellow lines) in patients with hereditary transthyretin amyloid nephropathy (ATTRv-N), after treatment with siRNA. Data were censored at the time of orthotopic liver transplantation, dialysis, loss to follow-up or death. ATTRv-N, ATTRv nephropathy; CKD-EPI, Chronic Kidney Disease - Epidemiology Collaboration formula; siRNA, small interfering RNA.

**Figure 4:**
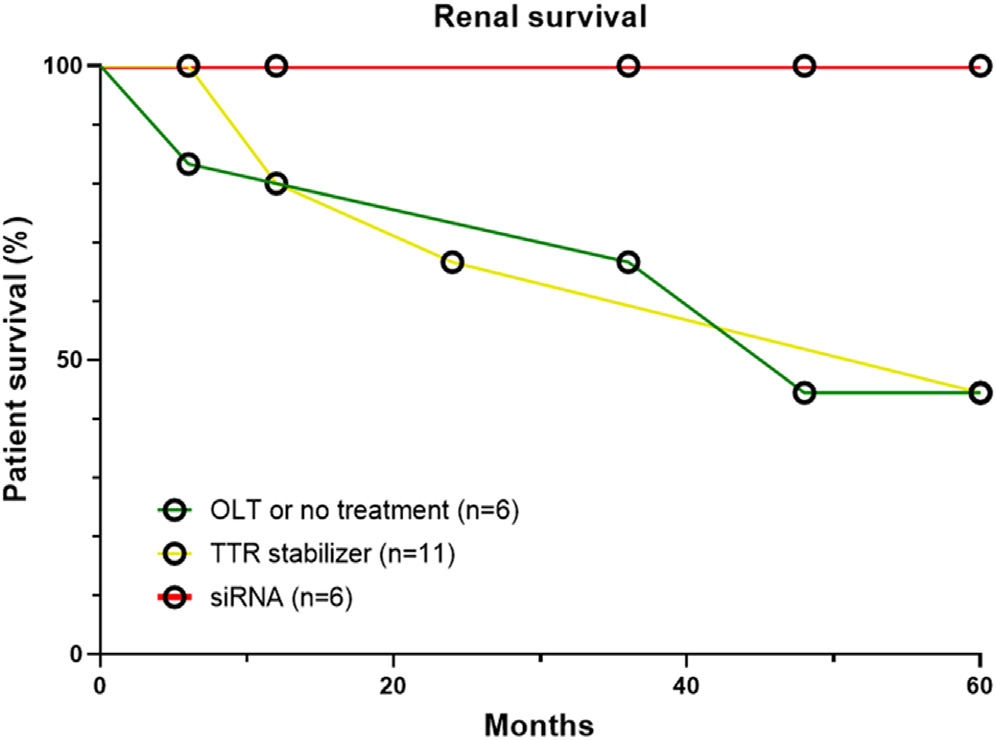
Renal survival in patients with ATTRv-N. Renal survival was assessed by a composite end point including an eGFR decline > 50% from the baseline and/or dialysis requirement, according to patients’ treatment: no treatment or orthotopic liver transplantation only (green line), TTR stabilizer only (yellow line) or siRNA (red line). ATTRv-N, ATTRv nephropathy; eGFR, estimated glomerular filtration rate; siRNA, small interfering RNA; TTR, transthyretin.

We next investigated the effect of treatments on renal outcomes from the diagnosis of ATTRv-N (Figure 1, Figure 2, Figure 3, Figure 4, and Table 3). Nine patients (39%) were on angiotensin-converting enzyme inhibitor or angiotensin II receptor blocker at baseline. All data were censored at the time of death, loss to follow-up, or OLT for patients on medical targeted therapies. Overall, 4 patients were treated exclusively with OLT (patients 1–4) and 2 did not receive any specific treatment (patients 5 and 6) (“group 1”). Eleven patients received tafamidis (patients 7–17) (“group 2”), and 6 received siRNA therapy (patients 18–23) (“group 3”). Four patients treated with tafamidis (patients 7 and 9–11) and 1 patient treated with siRNA (patient 18), had previously undergone OLT at the time of specific treatment initiation for ATTRv-N, including the domino liver recipient (patient 7). One patient (patient 21) treated with siRNA therapy had also received tafamidis followed by inotersen (an antisense oligonucleotide) before patisiran. Ten patients (44%) died during the follow-up as follows: 4 who underwent OLT or received no treatment (patients 1, 2, 4, and 5), 5 treated with tafamidis (patients 7, 10, 11, 16, and 17), and 1 treated with patisiran (patient 18).

**Table 3:** Risk factors associated with kidney function decline in ATTRv-N

Variables	eGFR decline > 50% from baseline / dialysis requirement		*P-*value
	Yes (*n* = 7)	No (*n* = 16)	
Age (yrs)	52 (35–67)	53 (42–65)	0.71
Female	5/7 (71)	11/16 (69)	0.99
LVEF < 45%	1/6 (16)	1/16 (6)	0.48
Bladder dysfunction	1/7 (14)	3/16 (19)	0.99
Age at first symptoms	34 (31-65)	45 (33-50)	0.99
Duration of symptoms before ATTRv-N diagnosis (mos)	2100 (714–6985)	1441 (365–2096)	0.24
Baseline eGFR < 30 ml/min per 1.73 m^2^	1/7 (14)	1/16 (6)	0.53
Baseline UPCR (mg/mmol)	180 (50–250)	100 (13–203)	0.31
Treatment groups			0.14
No treatment or OLT	3/7 (43)	3/16 (19)	0.32
TTR stabilizer	4/7 (57)	7/16 (44)	0.67
siRNA	0/7 (0)	6/16 (38)	0.16

ATTRv, hereditary transthyretin amyloidosis; ATTRv-N, ATTRv nephropathy; eGFR, estimated glomerular filtration rate by CKD-EPI (Chronic Kidney Disease Epidemiology collaboration); LVEF, left ventricular ejection fraction; OLT, orthotopic liver transplantation; TTR, transthyretin; UPCR, urine protein-to-creatinine ratio. Analyses of factors associated with renal survival were performed with Chi-square tests, Fisher exact tests, or *t* tests or Mann-Whitney tests, as appropriate. Values of *P* < 0.05 were considered statistically significant, all tests were 2-tailed.

Data are presented as *n* (percentage) of patients or median (interquartile range).

As shown in Figure 1, all patients from “group 1” (OLT or no treatment) had a progressive eGFR decline, and 3 of 6 required maintenance dialysis (after 25, 36, and 65 months from ATTRv-N diagnosis). Baseline significant proteinuria, present in 3 of 6 patients, remained stable throughout follow-up. Among patients from “group 2” (tafamidis), 8 or 11 (73%) showed a progressive decrease in eGFR, with 1 requiring maintenance dialysis. All patients with a previous OLT in this group had an eGFR decline over time. Significant proteinuria was more frequent in patients with eGFR decline (6/8, 75%) than in patients with stable eGFR (1/3, 33%), whereas left ventricular ejection fraction was quite similar between patients treated with tafamidis who had a stable versus a declining eGFR. Baseline significant proteinuria, present in 7 of 11 patients, mostly remained stable (patients 7, 10, 13, and 15) or increased (patient 8) during the follow-up (Figure 2). Patients 8 and 11, who reached end-stage kidney disease, received a combined liver and kidney transplantation. Only 1 patient (patient 17) displayed a decreased UPCR 6 months after the initiation of tafamidis along with an angiotensin-converting enzyme inhibitor, and had a stable eGFR, but died shortly thereafter. Finally, patients from “group 3” (siRNA therapy with patisiran or vutrisiran, with or without tafamidis) had a better renal outcome (Figure 1, Figure 3), with stabilization (patients 19, 21, and 23) or even improvement (patients 20 and 22) of eGFR over time, except in the OLT recipient (patient 18) who had a declining eGFR. There were 2 of 6, 3 of 11, and 4 of 6 patients of groups 1, 2, and 3, respectively, who were on angiotensin-converting enzyme inhibitor or angiotensin II receptor blocker, respectively. Overall, 7 patients (30%) reached the renal composite end point, none in the siRNA group, but no significantly associated risk factor was found by univariate analysis (Table 3). Renal survival was 44%, 44%, and 100% at 60 months for the 3 groups, respectively (*P* = 0.12). Renal survival was not statistically different between groups 1 and 2 (HR = 0.80, 95% confidence interval: [0.18–3.58], *P* = 0.99), between groups 1 and 3 (HR = 8.25, 95% confidence interval: [0.85–79.88], *P* = 0.08), and between groups 2 and 3 (HR = 5.66, 95% confidence interval: [0.74–43.43], *P* = 0.12) (Figure 4). Interestingly, UPCR was dramatically reduced in patients treated with siRNA therapy. Patient 20 (V30M variant), who presented with nephrotic syndrome and abundant glomerular amyloid deposition, responded quickly after the initiation of patisiran, with a > 50% reduction of UPCR at 6 months and a complete and sustained remission after 12 months, that persisted after vutrisiran introduction. Patient 19, who was initially treated with tafamidis for 2 years with a declining eGFR and persisting nephrotic syndrome, experienced a complete and sustained remission of proteinuria after the switch to patisiran, with a stabilization of eGFR. Patient 23 experienced a similar course, with a moderate decrease in proteinuria after 6 months of tafamidis, and a complete resolution after the introduction of patisiran. Patient 21 was diagnosed with ATTRv-N while receiving tafamidis. After a switch to inotersen, eGFR declined and UPCR increased after 3 months. Treatment with tafamidis was therefore resumed, along with the initiation of patisiran. A complete resolution of proteinuria was observed after 1 year and was maintained after a switch to vutrisiran. Finally, patient 22, who had a biopsy-proven ATTRv-N with stable CKD without significant proteinuria on tafamidis therapy for 2 years, experienced an improvement in eGFR after a switch to patisiran for worsening peripheral neuropathy.

## Discussion

The present study retrospectively describes the renal outcomes of a cohort of patients with ATTRv-N from 6 French rare disease referral centers. To the best of our knowledge, this is the largest series of patients treated for ATTRv-N, defined as histologically proven or very likely presence of renal TTR amyloid deposits. We found that siRNA therapy seems to be beneficial for the stabilization of kidney function and reduction of proteinuria.

Kidney involvement is common in patients with AA or AL amyloidosis and is typically characterized by high-grade proteinuria and a progressive decline in kidney function.^11^^,^^12^ Conversely, in ATTRv, kidney amyloid deposition remains an exceptional finding, although the first cases described by Andrade included patients with severe nephrotic syndrome.^3^^,^^13^ Therefore, most studies addressing the clinical spectrum of patients with ATTRv only reported eGFR without assessing proteinuria. Surprisingly, we previously observed that urinalysis was missing in approximately 41% of cases of our whole ATTRv cohort, highlighting a lack of awareness of kidney involvement in this disease.^5^ Two French independent studies reported that up to 30% of patients with ATTRv display CKD and up to 20% have significant proteinuria.^5^^,^^6^ This prevalence was quite similar in another cohort from Italy.^14^ In a large European collaborative study of 1181 patients with ATTR-related cardiopathy, microalbuminuria was observed in 48% of cases, and was correlated with a more severe cardiac phenotype and higher mortality rate.^15^ In this setting, kidney disease seems to be mostly dependent on indirect mechanisms reflecting the severity of cardiac dysfunction.^5^^,^^16^ Indeed, in a retrospective study of 232 patients, we found that the short-term natural decline of kidney function in ATTRv was unexpectedly fast, because the median estimated change in eGFR was approximately −7 ml/min per 1.73 m^2^ in the first year after diagnosis. Older age, lower left ventricular global longitudinal strain, and the V122I variant (associated with a prominent cardiac phenotype) were the main independent risk factors.^5^ Renal infarction is another potential outstanding cause of kidney injury, as documented in approximately 1 in 5 patients with amyloidosis, including patients with ATTRv and those with wild-type ATTR, by using ^99m^technetium-labeled dimercaptosuccinic acid renal scintigraphy.^17^ However, renal prognosis seems influenced by long-lasting proteinuria, suggesting kidney amyloid deposition at least in some cases.^6^^,^^18^ In a retrospective study of 30 patients with biopsy-proven ATTRv-N, we pointed out that direct kidney involvement may be underestimated, and can be present at an earlier stage, even in the absence of significant proteinuria, especially in patients carrying the V30M variant.^4^ Other series of biopsy-proven ATTRv-N highlighted the presence of amyloid deposits without significant impairment of kidney function.^19^, ^20^, ^21^ Furthermore, extraglomerular amyloid deposition, particularly in the medullary interstitium, seems to be more frequent than in other types of systemic amyloidosis.^4^ Thus, the absence of proteinuria does not rule out the existence of amyloid nephropathy.^4^^,^^19^ Altogether, direct or indirect kidney involvement of ATTRv may be more frequent than expected. Describing renal outcomes in this population is thus a relevant issue for the nephrology community, with the availability of novel and well-tolerated targeted therapies with striking beneficial effects on neurological and cardiac symptoms.

Liver transplantation has long been considered a first-line treatment for ATTRv, in patients fit enough to undergo surgery. However, OLT is associated with a high risk of secondary and multifactorial CKD,^22^ favored by postsurgical complications such as sepsis and hypovolemia and long-term use of calcineurin inhibitors. Moreover, kidney amyloid deposits have been shown to remain stable even after 2 years from OLT and progression of nephropathy is theoretically possible because of continued amyloid deposition from wild-type TTR.^20^^,^^23^ Indeed, in our study, we found that all patients with ATTRv-N who underwent OLT alone or before other specific therapies, had a progressive decline in eGFR over time, most reaching end-stage kidney disease. As a comparison, a French cohort found that 52% of the liver transplant recipients had CKD after 5 years of follow-up, defined by an eGFR < 60 ml/min per 1.73 m^2^, as compared with 78% (7/9) of our OLT patients.^24^ However, because of the previously cited confounding factors, we do not think that OLT is not beneficial to some extent for kidney function by suppressing TTR production, and our work was not meant to exclude OLT from the therapeutic strategy, because it might still be helpful in some cases for neurologic and cardiac symptoms.

Tafamidis, a TTR stabilizer, was the first approved drug that deeply changed the outcomes of patients with ATTRv, with beneficial effects on cardiac outcomes and stabilization of neurological symptoms, and a good safety profile, without renal toxicity.^25^ In a *post hoc* analysis of the ATTR-ACT study, the use of tafamidis reduced eGFR decline and was associated with higher rates of improved eGFR and better CKD staging over 30 months, compared with placebo.^26^ This difference became significant after 18 months of treatment. Considering that most patients of this study did not have biopsy-proven ATTRv-N, the favorable effects of tafamidis on kidney function may partly reflect the improvement of cardiac function. Few case reports also suggest a favorable effect of tafamidis on proteinuria in ATTRv-Rocha *et al.*^27^ reported 9 Portuguese patients with normal eGFR, including 4 with mild proteinuria, and 2 with biopsy-proven ATTRv-N, who maintained a stable eGFR over 36 months under tafamidis treatment, with moderate reduction in proteinuria. Ferrer-Nadal *et al.*^28^ described a woman with ATTRv (V30M variant) presenting with multisystem involvement and nephrotic syndrome (without histological confirmation of ATTRv-N). Proteinuria decreased from 4.2 g/d to 1.6 g/d after 1 year on tafamidis, and remained stable for another year, with stable eGFR value. Ikeda *et al.*^29^ reported a 63-year-old woman, with a biopsy-proven ATTRv-N (V30M variant), in whom proteinuria was dramatically reduced with tafamidis, but with persistent hypoalbuminemia and eGFR decline after 1 year.^29^ These 2 cases were the first to suggest the efficacy, albeit incomplete, of a targeted therapy on renal disease in ATTRv. However, in the present cohort, several patients with biopsy-proven ATTRv-N developed renal symptoms while on tafamidis therapy, and initiation of tafamidis failed to reduce proteinuria or to stabilize eGFR in most cases, even in non-OLT patients. This is consistent with previous observations suggesting a lack of effectiveness of TTR stabilizers in some patients with mixed phenotypes. Our study indicates that the clearance of kidney amyloid deposits in ATTRv-N is slow with tafamidis, which had beneficial effect in the ATTR-ACT trial that might rather result from improved cardiac function.

Until now, gene silencers in ATTRv, including the siRNAs, patisiran and vutrisiran, and the antisense oligonucleotide, inotersen, are the only therapies to suppress TTR production. All have shown efficacy on cardiac and neurological outcomes. Inotersen was associated with a higher incidence of adverse renal events in the NEURO-TTR trial, including rapidly progressive glomerulonephritis in 3% of patients.^30^ Law *et al.*^31^ also reported a 30-year-old woman with ATTRV30M, presenting with nephrotic syndrome 7 months after initiation of inotersen, and focal segmental glomerulosclerosis lesions on kidney biopsy with only scanty glomerular TTR amyloid deposits. Discontinuation of inotersen resulted in complete resolution of the nephrotic syndrome. Although the open-label extension study of the NEURO-TTR trial did not confirm this risk of nephrotoxicity, inotersen is currently not recommended for patients with altered eGFR (< 45 ml/min per 1.73 m^2^) or proteinuria (> 1 g/g). In contrast, patisiran has not been associated with renal adverse events. In an Italian case series of 17 patients with ATTRv, trajectories of kidney function remained stable after initiation of gene silencers, either with inotersen or patisiran, with possibly slower eGFR decline in the patisiran group.^32^ Here, we detailed 5 cases of biopsy-proven ATTRv-N, successfully treated with patisiran. Four patients presented with nephrotic-range proteinuria, 3 were resistant to tafamidis. Patisiran led to a fast, complete and sustained remission of proteinuria after 1 year, which was safely maintained by vutrisiran. In 1 patient with CKD, switch from tafamidis to patisiran led to an unexpected improvement in eGFR. However, this patient did not have severe kidney involvement or proteinuria, suggesting that this beneficial effect was partly due to the improvement of cardiac function. Indeed, significant regression of cardiac lesions by magnetic resonance and bone scintigraphy cardiac uptake has been described with patisiran.^33^^,^^34^

Our study has several limitations other than its retrospective nature. First, cardiac function was not repeatedly monitored for all patients, and the effect of progressive heart dysfunction on renal outcomes could not be excluded, at least in some cases. However, because only less than 10% of patients had decreased left ventricular ejection fraction at baseline, we hypothesize that cardiorenal syndrome was probably not the main contributor to eGFR decline. Second, this study suggests a superiority of patisiran over tafamidis among patients with high-grade proteinuria; however, because of the small sample size, we cannot rule out a positive effect of tafamidis in patients in whom tafamidis was associated with patisiran, or in the long-term in patients with milder renal disease, as shown in previous case series.^27^, ^28^, ^29^ Third, the use of nephroprotective drugs, renin-angiotensin-aldosterone system and sodium-glucose transport protein 2 inhibitor, was uneven over the years in our patients, and not always reported in medical charts. Further studies will help to assess the efficacy of these therapies on kidney outcomes in ATTRv patients; however, recent data suggest that sodium-glucose transport protein 2 inhibitors may be a relevant option for slowing eGFR decline.^16^^,^^35^ Finally, 2 of the 3 patients without biopsy-proven ATTRv-N displayed a V122I variant, not usually associated with direct kidney involvement, and were of African ancestry. Whether other factors, such as *APOL1* variants, might have contributed to kidney disease remains unknown.

## Conclusion

Our study is the first to provide data about renal outcomes under targeted therapies in ATTRv-N; and suggests that siRNA therapy could be particularly effective for stabilizing kidney function and treating high-range proteinuria. The beneficial effect of tafamidis on kidney function cannot be ruled out, especially through improvement of cardiorenal syndrome over the long term. We recommend systematic screening of eGFR and UPCR in patients with ATTRv, and the broader use of kidney biopsy to document ATTRv-N, especially in clinical trials, to better assess the effectiveness of targeted therapies in ATTRv-associated renal disease.

## Disclosure

JD received lecture and/or travel fees from Alnylam, Chiesi, and Sandoz. NJ-C and JS received lecture fees from Alnylam. VA received consulting fees from Addmedica, Sanofi Genzyme, Vifor, Alnylam, and AstraZeneca. TD received consulting fees and/or research grants from Alnylam, Bayer, Ionis, Janssen, Akcea, GSK, Pfizer, Prothena, Novartis, and Neurimmune. FB received consulting fees and/or research grants from Janssen, Novartis, Prothena, Amgen, Sanofi, GSK, and Vifor. AE-L received consulting and/or lectures fees from Alnylam and Pfizer. AH received lectures fees from Pfizer. PD received support for attending meetings from Alnylam and Pfizer. These fees were all received outside of this project. All the other authors declared no competing interests.

## Author Contributions

The research idea was developed by JD. Diagnosis and follow-up of patients were done by all authors. Data acquisition was done by JD. Data analysis or interpretation was done by JD, JS, MZ, NJC, and VA. Supervision or mentorship was by MZ, NJC, and VA.
